# Theoretical Specific Capacity and Metal Ion Diffusion Pathway of NiMoO_4_ Microspheres for Hybrid Supercapacitors

**DOI:** 10.1002/smll.202500080

**Published:** 2025-02-24

**Authors:** Digambar S. Sawant, Sandesh V. Gaikwad, Akash V. Fulari, Mani Govindasamy, Shrinivas B. Kulkarni, Deepak P. Dubal, Gaurav M. Lohar

**Affiliations:** ^1^ Department of Physics The Institute of Science Dr. Homi Bhabha State University Madam Cama Road Mumbai 400032 India; ^2^ Department of Physics Lal Bahadur Shastri College of Arts Science and Commerce Satara 415002 India; ^3^ Symbiosis Centre for Nanoscience and Nanotechnology Symbiosis International (Deemed University) Pune 412115 India; ^4^ Research Center for Intelligence Medical Devices Ming Chi University of Technology New Taipei City 243303 Taiwan; ^5^ School of Chemistry & Physics Centre for Material Science Queensland University of Technology Brisbane QLD 4000 Australia

**Keywords:** density functional theory, hybrid supercapacitor, nickel molybdate microspheres, theoretical specific capacity

## Abstract

Transition metal molybdates are one of the most prominent materials for energy storage devices. The present investigation establishes a strong correlation between the structure and electrochemical performance of NiMoO_4_ through Density Functional Theory (DFT). Initially, the NiMoO_4_ microspheres are directly deposited on nickel foam using a hydrothermal method by tuning experimental parameters. When employed as electrode materials, the NiMoO_4_ microspheres deliver a specific capacity of 168.9 mAh g^−1^ at 1 A g^−1^. In addition, the material retains 80% capacity over 7000 charge‐discharge cycles with 98.3% coulombic efficiency, implying its excellent stability. DFT calculations are used to determine specific capacity and potassium ion diffusion for 5 layers of [110] planes of NiMoO_4_. The potential energy landscape is created for [110] plane using the potassium atom minimum hopping algorithm and atomic simulation environment. The DFT results clearly align with the theoretical capacity of 203 mAh g^−1^ close to the experimental results. A hybrid supercapacitor (HSC) is also developed with NiMoO_4_//AC cell delivers a specific energy of 56.3 Wh kg^−1^ at a specific power of 421 W kg^−1^ with negligible capacity loss over 15 000 cycles. This investigation offers the development of battery‐type electrodes for hybrid supercapacitors using the fundamental understanding of ion‐diffusion in the materials’ structure.

## Graphical abstract

This study explores the theoretical specific capacity and metal ion diffusion pathway of NiMoO₄ microspheres, highlighting their potential as battery‐type electrodes for hybrid supercapacitors. With an extended operational voltage, high energy density, and long cycling stability, NiMoO₄ offers a promising solution for next‐generation energy storage systems.

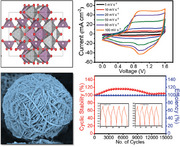

## 1 Introduction

Global energy consumption has risen, fueled by factors like population growth, industrial expansion, and rapid technological progress. Amid concerns over climate change and the gradual exhaustion of fossil fuel reserves, there is a global shift toward renewable energy sources, particularly solar and wind. However, the variability of these sources presents challenges for maintaining a stable and reliable power grid. To keep up with growing energy needs while moving toward a sustainable, low‐carbon future, efficient energy storage solutions have become essential. These storage technologies play a vital role by capturing surplus energy when production is high and releasing it when demand peaks, covering a range from traditional batteries to innovative, next‐generation systems.^[^ ^1^, ^2^, ^3^, ^4^, ^5^ ^]^ Energy storage devices such as supercapacitors and batteries play a crucial role in bridging the gap between intermittent renewable energy sources and the continuous energy demands of society. Supercapacitors have been the most important class of energy storage devices. A supercapacitor holds significance for its swift charge‐discharge capability, extended cycle lifespan (>100 000 cycles), and superior power density in contrast to rechargeable batteries, while also boosting a higher energy density compared to conventional capacitors.^[^ ^6^, ^7^, ^8^, ^9^ ^]^

Hybrid supercapacitors represent a versatile class of energy storage devices that combine the strengths of both electrochemical double‐layer capacitors (EDLC) and pseudocapacitors. By integrating different materials for the positive and negative electrodes typically a carbon‐based material for the EDLC behavior and a metal oxide or conducting polymer for the pseudocapacitive or battery‐like behavior.^[^ ^10^, ^11^, ^12^, ^13^ ^]^ The HSC achieves a balance between high energy density and high power density. This combination allows them to deliver higher energy storage capacity than traditional EDLC while maintaining the rapid charge‐discharge capabilities and long cycle life characteristic of supercapacitors. As a result, HSC are particularly well‐suited for applications requiring both quick bursts of energy and sustained energy output, such as in electric vehicles, portable electronics, and renewable energy systems. The urgent need to enhance electrochemical energy storage technologies in terms of energy capacity, power capability, and cycling life has driven the development of HSC. These innovative HSC devices integrate the advantages of both rechargeable batteries and supercapacitors into a single system.^[^ ^14^, ^15^ ^]^ In the current year most of the researchers have chosen transition metal oxides, metal sulfides, and metal molybdates like as nickel cobalt oxide (NiCo_2_O_4_),^[^ ^16^ ^]^ nickel cobalt sulfides (NiCo_2_S_4_),^[^ ^17^ ^]^ manganese molybdate (MnMoO_4_),^[^ ^18^ ^]^ and nickel molybdate (NiMoO_4_)^[^ ^19^ ^]^ have been mostly used as battery‐type material due to their strong redox reactions and high energy storage capacity. Among those, NiMoO_4_ has garnered attention due to the heightened electrochemical reactivity of nickel atoms and the outstanding conductivity of molybdenum atoms when compared to other metal oxides.^[^ ^20^, ^21^, ^22^ ^]^ The synthesis of NiMoO_4_ microspheres directly deposited on nickel foam using the hydrothermal method offers significant advantages over other morphologies and chemical synthesis methods. The direct deposition onto nickel foam eliminates the need for binders or conductive additives, reducing interfacial resistance and enhancing charge transfer efficiency. Also, the direct deposition strategy ensures better cycling stability, faster charge‐discharge performance, and higher specific capacity due to efficient utilization of active sites and enhanced redox reactions.^[^ ^23^ ^]^ The microsphere morphology provides a hierarchical structure with high surface area and interconnected pores, facilitating efficient ion diffusion, rapid electrolyte penetration, and a large number of active sites for electrochemical reactions. This structure outperforms other morphologies, such as nanorods or nanoplates, by offering better stability and uniform distribution of active sites. Additionally, the hydrothermal method ensures strong adhesion of the NiMoO_4_ microspheres to the nickel foam, which improves mechanical stability and minimizes active material detachment during prolonged cycling, enhancing durability.^[^ ^24^ ^]^ The literature (**Table** **1**) suggested that the morphology of NiMoO_4_ microspheres significantly impacts its electrochemical performance with nanosheets and nanoflowers showing superior specific capacitance and cyclic stability. However, there is a lack of research on hybrid materials and in‐depth mechanistic studies that could further enhance performance. DFT is a powerful computational tool that plays a crucial role in the development and optimization of supercapacitor applications. Its importance lies in its ability to provide a detailed understanding of the electronic structure specifically the density of states (DOS) and band structure, charge distribution, and surface interactions of materials at the atomic level. This includes insights into key properties like specific capacity, ion adsorption energies, and the diffusion pathways of ions within electrode materials, all of which are critical for enhancing energy storage performance.^[^ ^25^, ^26^, ^27^ ^]^ By performing DFT calculations to obtain the electronic structure of NiMoO_4_, we can accurately evaluate its energy storage performance. Understanding the relationship between the electronic structure and surface diffusion processes is crucial for supercapacitive materials where surface reactions and kinetics play a significant role.^[^ ^28^ ^]^

**Table 1:** Electrochemical performances of transition metal molybdate based materials.

Electrode material	Morphology	Specific capacitance [F g^−1^]	Calculated specific capacity [mAh g^−1^]	Cyclic stability	Electrolyte	Refs.
NiMoO_4_	Nanoflowers	1092	97.2	91% after 2000 cycles	6 m KOH	[22]
NiMoO_4_	Nanospheres	974	43.5	74.5% after 2000 cycles	3 m KOH	[40]
NiMoO_4_	Nanosheets	864	13.9	71% after 1000 cycles	2 m KOH	[41]
NiMoO_4_	Nanosheets	1221	71.8	89.2 over 10 000 cycles	2 m KOH	[42]
CoMoO_4_	Nanoflakes	614	87.5	89.2% after 2000 cycles	2 m KOH	[43]
CoMoO_4_	Nanorods	286	138.8	97.5% after 2000 cycles	2 m KOH	[44]
CoMoO_4_	Nanoplates	133	25.5	84% after 1000 cycles	2 m KOH	[45]
FeMoO_4_	Cuboids	493	46.3	‐	6 m KOH	[46]
MnMoO_4_	Nanorods	836	44.4	84% after 3000 cycles	1 m KOH	[47]
NiMoO_4_	Microspheres	1520	168.9	80% over 7000 cycles	2 m KOH	This work

This work is focused on the development of a hybrid supercapacitor based on NiMoO_4_ materials by understanding the ion‐diffusion and transport mechanisms in the structure of electrodes using DFT theory. First, the work systematically tunes the hydrothermal reaction parameters for the synthesis of NiMoO_4_ and achieves optimal electrochemical performance. The study revealed that NiMoO_4_ microspheres exhibit excellent structural and electrochemical properties as compared to the other morphologies. The DFT calculations provide a comprehensive picture of the NiMoO_4_ system, enabling a deeper understanding of its capacitive and surface‐related characteristics that help to improve its electrochemical performance. Our approach uniquely combined structural relaxation, self‐consistent field calculations, DOS analysis, and band structure calculations to provide a holistic view of NiMoO_4_ electronic properties. We employed the PBEsol exchange‐correlation functional with carefully chosen pseudopotentials and included spin polarization to account for the complex d‐orbital interactions. Our calculations revealed NiMoO_4_ to be a narrow‐gap semiconductor with a band gap of 2.68 eV, consistent with previous studies. Furthermore, we extended our analysis to include work function calculations for a 5‐layered slab oriented along the [110] plane, finding that 7.1 eV is required to eject an electron. For NiMoO_4_ supercapacitor materials, first ever we calculated the specific capacity for NiMoO_4_ using DFT, and the specific capacity for [110] plane was found to be 203 mAh g^−1^. These findings provide crucial insights into NiMoO_4_ electronic structure and its potential applications in supercapacitors.

## 2 Results and Discussion

**Figure 1:**
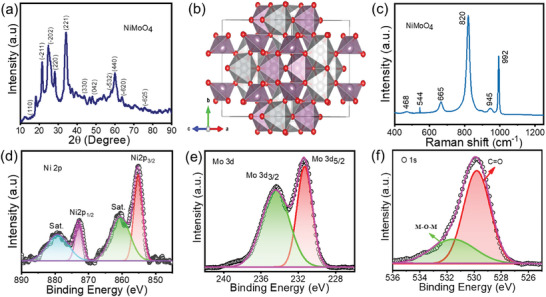
a,b) XRD patterns of optimized NMO‐6 h sample with its polyhedron model. c,d) Raman and XPS spectra of optimized NMO‐6 h sample with magnified plots of d) Ni 2p, e) Mo 3d f) O 1s, respectively.

The material formation, crystal structure, and crystalline grain size of nanoparticles were determined by using the X‐ray diffraction (XRD) technique. The optimized NiMoO_4_ material sample without nickel foam substrate and obtained the XRD pattern of NiMoO_4_ material in **Figure** **1**a. The diffraction peaks were indexed to the monoclinic phase having space group of C2/m with their lattice parameters a = 9.566A°, b = 8.734A°, c = 7.649 A° and matched well with JCPDS card no. 01‐086‐0361. The crystal structure of NiMoO_4_ exists mainly in two phases (α and β‐phase). α‐phase shows the monoclinic structure while β‐phase shows the orthorhombic crystal structure.^[^ ^29^ ^]^ The polyhedron model of α‐phase monoclinic crystal structure of NiMoO_4_ is shown in Figure 1b. The monoclinic polyhedron model of NiMoO_4_ vividly illustrates the coordination environment of Ni and Mo atoms within the crystal structure. In this model, the Ni atoms are depicted in octahedral coordination, where each Ni atom is surrounded by six O atoms, forming NiO_6_ octahedra. These octahedra are typically represented in a distinct color, such as gray, to differentiate them from other polyhedra. On the other hand, Mo atoms exhibit tetrahedral coordination, with each Mo atom surrounded by four O atoms, forming MoO_4_ tetrahedra, often shown in violet. This arrangement results in a network where the NiO_6_ octahedra and MoO_4_ tetrahedra are interconnected through shared oxygen atoms, maintaining the structural integrity of the crystal. The monoclinic crystal system is characterized by three unequal axes (a, b, and c) with one angle β differing from 90°, while the other two angles α and γ are 90°. This asymmetry is reflected in the polyhedral model, providing insight into the unique lattice parameters and angles specific to the monoclinic NiMoO_4_ structure. XRD pattern of NiMoO_4_ samples at a different reaction time of 4, 5, 6, and 7 h are shown in Figure S1 (Supporting Information), and the XRD pattern of NiMoO_4_ samples at different calcination temperatures of 350, 400, and 450 are shown in Figure S2 (Supporting Information). The three high‐intensity diffraction peaks at 44.5°, 51.8,° and 76.5° of nickel foam which is dominated the NiMoO_4_ material peaks. To determine the structural functional groups of the NiMoO_4_ material, the optimized NMO‐6hr sample was analyzed by Raman spectroscopy, and the Raman spectra of NiMoO_4_ material are shown in Figure 1c. The eminent highly intense sharp Raman band was observed at 820 cm^−1^ and the weaker Raman band at 992 and 945 cm^−1^. These Raman peaks at 992, 945, and 820 cm^−1^ are attributed Mo─O bonding of symmetric and antisymmetric stretching modes.

X‐ray photoelectron spectroscopy (XPS) is employed for the examination of the chemical composition and oxidation states of NiMoO_4_ microsphere materials. XPS reveals the interaction between Ni and Mo, aiding in optimizing the material's composition for enhanced energy storage capacity and cycling stability. The total wide scan XPS survey spectrum of optimized NMO‐6 h material is composed of Ni, Mo, and O components with corresponding binding energy is shown in Figure S3 (Supporting Information). The narrow scan survey spectrum of Ni 2p is observed in Figure 1d. The two deconvolution peaks at binding energy 855.48 and 872.88 eV show the Ni 2P_3/2_ and Ni 2P_1/2_ energy levels, which indicate Ni^2+^ oxidation state. The other peaks observed at 860.45 and 877.85 eV are associated with satellite peaks of Ni 2P_3/2_ and Ni 2P_1/2_ energy levels and the spin‐energy separation value of Ni element between the two‐energy level is 17.4 eV. The Mo 3d core level XPS spectrum is observed in Figure 1e. In that spectrum, two peaks found at 231.3 and 234.5 eV were ascribed for Mo 3d_5/2_ and Mo 3d_3/2_. The splitting energy levels divulge Mo^6+^ oxidation state with 3.2 eV energy separation between them. In O 1s spectrum displayed in Figure 1f, the given peaks are broken down into two primary peaks at 529.8 and 531.4 eV and correspond to C═O and M─O─M metal oxygen bonding.^[^ ^30^ ^]^

**Figure 2:**
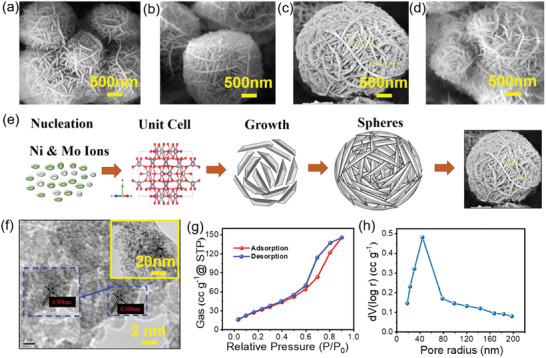
a–d) SEM images of NMO‐4, 5, 6, and 7 h, respectively. e) Growth mechanism of NiMoO_4_ microspheres. f) TEM images of NMO‐6 h sample at various magnifications (inset). g,h) Nitrogen adsorption/desorption isotherms and BJH pore size distribution of NMO‐6 h sample.

The surface morphology of the time‐series NiMoO_4_ samples was examined at various magnifications using field emission scanning electron microscopy (FESEM). **Figure** **2**a–d displays the FESEM images of the NMO‐4, NMO‐5, NMO‐6, and NMO‐7 h samples, synthesized by varying the reaction time using the hydrothermal method. The FESEM study reveals the formation of microspheres. Initially, at a reaction time of 4 h, microspheres with a diameter of ≈7µm are observed (Figure 2a). These microspheres are composed of nanorods. As the reaction time is increased to 5 h, the size of the microspheres decreases to ≈5 µm (Figure 2b). Further increasing the reaction time to 6 h results in microspheres with a diameter of ≈2.6 µm (Figure 2c). This size of microspheres is very low in size to the other time series samples and it also well‐developed microspheres from the agglomeration of the nanorods. Finally, at a reaction time of 7 h, the microspheres disappear, and the observed structures have a diameter of ≈6 µm (Figure 2d). The overall morphology of the NiMoO_4_ samples synthesized at different reaction times is clearly characterized by the presence of numerous microspheres with an average diameter of ≈2.6 µm at lower magnifications. Higher magnification reveals that these microspheres possess a hierarchical structure, composed of nanorods with an average diameter of ≈31.1 nm. Figure S4 (Supporting Information) provided SEM images of NiMoO_4_ microspheres on nickel foam at lower magnification.

The morphology appears to consist of a spherical structure covered with nanorods. The growth mechanism of NiMoO_4_ microspheres is displayed in Figure 2e. The growth process begins with the nucleation of NiMoO_4_ particles in a solution‐based synthesis by hydrothermal method. In the initial stage the precursors nickel nitrate and sodium molybdate produce the nickel and molybdate $\left(Ni^{2+}\;\mathrm{and}\;{\mathrm{MoO}}_{4}^{2-}\right)$ ions dispersed in a solution. These precursor ions are the building blocks that will eventually form the NiMoO_4_ nanostructures through nucleation and growth processes. In the second nucleation stage, the precursors react in the solution, small nuclei begin to form. These nuclei serve as seeds for further growth. During this phase, the reaction conditions (temperature, concentration of precursors, and time) dictate the growth direction, resulting in the formation of elongated nanostructures. The unit cell suggests that the formation of ordered NiMoO_4_ structures begins at this stage, which is crucial for determining the orientation and growth direction along in *Z* axis of the nanorods. The third stage demonstrates the development of individual nanorods. Once nucleation has occurred, these nanorods start growing in an anisotropic manner. The anisotropic growth of the rods could be driven by different surface energies of the crystal facets. As growth continues, these nanorods self‐assemble into larger, spherical agglomerates. This self‐assembly could be facilitated by van der Waals forces, electrostatic interactions, or solvent evaporation during the synthesis process.^[^ ^31^, ^32^ ^]^ The growth direction is determined by the crystallographic structure, with some crystal faces growing faster than others. This stage results in the formation of elongated nanostructures, which are the fundamental components of the final microsphere. The fourth stage shows the self‐assembly of nanorods are into a sphere like arrangement. These nanorods start to aggregate and arrange themselves in a layered or spiral fashion around a central point. The nanorods arrange themselves to minimize surface energy, leading to the observed spherical morphology. During the later stages of the reaction, smaller particles or nanorods may dissolve and redeposit onto the larger spheres, a process known as Ostwald ripening.^[^ ^33^, ^34^ ^]^ This further refines the structure, leading to more uniform and well‐defined spherical agglomerates of nanorods. The final stage depicts the completed NiMoO_4_ microsphere. The nanorods have completely assembled into a spherical structure, as observed in the FESEM image. The growth mechanism likely involves a combination of nucleation, oriented growth, self‐assembly, and Ostwald ripening, resulting in the observed spherical morphology. The densely packed nanorods create a hierarchical structure with a high surface area, which is advantageous for applications in electrochemical energy storage. Also, we have studied the effect of different calcination temperatures on morphology of optimised NMO‐6 h sample is explained and shown in Figure S5 (Supporting Information).

The transmission electron microscope (TEM) is employed for visualizing the internal structure, crystallographic property, morphology, and composition of materials. The TEM images are shown in Figure 2f. The Inset Figure shows the higher magnification TEM image of the optimized NMO‐6 h sample and lattice fringes showed a spacing of 0.30 nm.

The Brunauer‐Emmett‐Teller (BET) analysis of NiMoO_4_ was conducted to determine its surface area and pore characteristics. Employing a gas adsorption analyzer under controlled conditions of temperature and pressure, adsorption isotherms were obtained to characterize the material's porosity. The optimized sample NMO‐6 h was used for the BET analysis. The resulting isotherms exhibited characteristic features, allowing for the calculation of the BET surface area. As illustrated in Figure 2g, the isotherm displayed type IV isotherm and H1 hysteresis in accordance with the IUPAC categorization. The type H1 hysteresis loop is frequently connected with porous materials containing well‐defined cylindrical‐like pore channels or agglomerates of nearly homogeneous spheres of the NiMoO_4_ material.^[^ ^35^ ^]^ A small hysteresis loop is detected on this type IV isotherm between relative pressures of 0.5 and 1.0. The calculated surface area of NMO‐6 h sample was found to be 117.6 m^2^ g^−1^. Additionally, pore size distribution analysis was performed using the Barrett‐Joyner‐Halenda (BJH) method, revealing the distribution of pore size and total pore volume obtained are 40.2 nm and 0.2 cc g^−1^, respectively within the material is shown in Figure 2h. This analysis elucidated the abundant accessible surface area and mesoporous nature of NiMoO_4_, which are advantageous for enhancing electrolyte accessibility and ion diffusion in supercapacitors, thereby potentially improving their energy storage performance. These findings underscore the potential of NiMoO_4_ as a promising electrode material for high‐performance supercapacitors.

### 2.1 Electrochemical Investigation of NiMoO_4_ Electrodes

**Figure 3:**
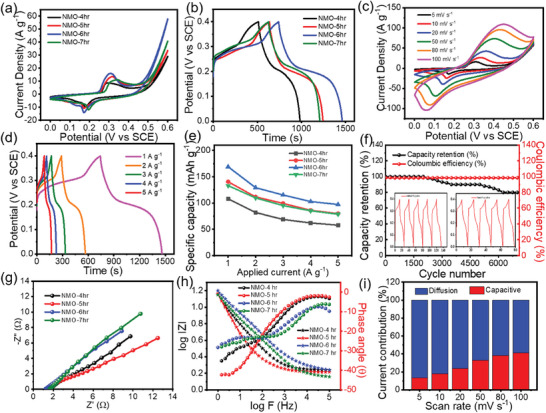
a,b) The comparative CV and CD curves of NMO‐4, NMO‐5, NMO‐6, and NMO‐7 h electrodes at 5 mV s^−1^ and 1 A g^−1^, respectively. c,d) CV and GCD curves of NMO‐6 h electrode, respectively. e) Variation of specific capacity as a function of the current density. f) Capacity retention and coulombic efficiency of NMO‐6 h electrode. Inset shows initial and last 5 cycles. g,h) Nyquist plots of NMO electrodes and Bode plots, respectively. i) Total contribution of capacitive and diffusion‐controlled charge storage.

**Figure** **3**a,b depicts the overall electrochemical study of time series. Figure 3a shows the cyclic voltammetry (CV) curves of NMO‐4, NMO‐5, NMO‐6, and NMO‐7 h electrodes at a minimum scan rate of 5 mV s^−1^_._ Figure S6a–c (Supporting Information) represents the CV curves of NMO‐4, NMO‐5, and NMO‐7 h at different scan rates (5‐100 mV s^−1^). During the CV process, the redox transitions are associated with faradaic reactions, which significantly enhance charge storage capacity. Specifically, the oxidation of Ni^2+^ to Ni^3+^ and the subsequent reduction back to Ni^2+^ are accompanied by the reversible uptake and release of hydroxide ions (OH^−^) from the electrolyte. This redox activity is typically represented by the reaction as *Ni* ^2 +^ +*OH* ^−^↔ *Ni* ^3 +^ +*e* ^−^. These reactions contribute to the pseudocapacitive behavior of NiMoO_4_, as they occur on the surface or near‐surface regions of the material, allowing rapid electron and ion transfer. Consequently, the Ni^2+^/Ni^3+^ transitions significantly influence the overall energy storage mechanism, delivering high specific capacity and superior electrochemical performance.^[^ ^36^ ^]^ Figure 3b represents the galvanostatic charge discharge (GCD) curves of NMO‐4, NMO‐5, NMO‐6, and NMO‐7 h electrode at a minimum current density of 1 A g^−1^_._ Figure S7a–c (Supporting Information) represents the GCD curves of NMO‐4, NMO‐5, and NMO‐7h at different current densities (1–5 A g^−1^). The CV and GCD graphs of optimized sample NMO‐6 h are depicted in Figure 3c,d. Figure 3e shows the specific capacity value of NMO‐4, NMO‐5, NMO‐6, and NMO‐7 h at different current densities (1‐5 A g^−1^). The charge discharge mechanism of NiMoO_4_ is explained in Equation S1 (Supporting Information). The maximum specific capacity was calculated from GCD curves for NMO‐4, NMO‐5, NMO‐6, and NMO‐7 h electrodes are 107.9, 140.5, 168.9, 133.1 mAh g^−1^ from the Equation (S2, Supporting Information) at 1 A g^−1^ respectively. Table S1 (Supporting Information) shows the calculated specific capacity values of the different samples with their discharging times from Equation (S2, Supporting Information).

The Equation (1) shows the relation of predicted specific capacitance (PSc) with respect to actual capacitance (ASc) in F g^−1^. Equation (1) is an output of machine learning (ML) analysis to understand the predicted specific capacitance for molybdate material. This is true for metal molybdate‐based supercapacitors.^[^ ^7^ ^]^ From the electrochemical analysis, the specific capacitance value is calculated from the traditional method and the calculated specific capacitance is 1520 F g^−1^. Now, the specific capacitance is calculated from experimental data mentioned in the manuscript and this specific capacitance value is considered as ASc. The PSc value for NiMoO_4_ sample is calculated from ML output. In this case, the ASc value is 1520 F g^−^^1^ and the PSc value is calculated as 1489 F g^−1^. The Equation (1) is modified using Equation (S3, Supporting Information) and we have calculated the predicted specific capacity (PScapacity) in mAh g^−1^.

(1) $$PSc\left(Fg^{-1}\right)=0.85\;ASc+197.84$$

(2) $$\mathrm{PSca}\mathrm{paci}\mathrm{ty}\left(\mathrm{mAh}g^{-1}\right)=\frac{\mathrm{PSc}\left(Fg^{-1}\right)\times \Delta v}{3.6}$$

From the electrochemical study we calculated a specific capacity value is 168.9 mAh g^−1^ and the corresponding PScapacity is 165.5 mAh g^−1^. These Equations (1) and (2) are very useful in the upcoming molybdate‐based material analysis.

From the supercapacitor analysis of various time and temperature series, we found that the NiMoO_4_ electrode prepared with a reaction time of 6 h, reaction temperature of 160 °C, and calcination at 400 °C exhibited superior electrochemical performance compared to other electrodes. The optimized NiMoO_4_ sample demonstrated excellent cycle stability, retaining 80% of its initial capacity over 7000 charge‐discharge cycles at 15 A g⁻^1^ with a coulombic efficiency of 98.3% (Figure 3f). The higher columbic efficiency shows that the ratio of time required to charge and discharge of the electrode material was the same. So, the columbic efficiency of the electrode material was constant from the initial to a final number of cycles. Due to electrolyte decomposition, material aging, ambient temperature, and different charge‐discharge current rates, the cyclic stability performance of the material gradually decreases.^[^ ^37^ ^]^ Figure S8a (Supporting Information) displays that the XRD and there are no significant changes observed in XRD. To understand the change in morphology after stability have studied using SEM (Figure S8b,c, Supporting Information). From SEM images it observed that there are no any significant changes in morphology. However, the nanorods present in microspheres are observed to collapse at a certain level. The deterioration of nanorods on microspheres is responsible for destroying the better solid–liquid interface. During charge discharge study, we observed that after 7000 cycles stability is decreased. It indicates that the nanorods on the microspheres are significantly destroyed after 7000 cycles. Also, the electrochemical characterizations indicate that the performance of the NMO‐6 h sample deteriorates during the charging and discharging process after 7000 cycles. The decrease in CV current (Figure S8d, Supporting Information), decreased discharging time (Figure S8e, Supporting Information) and increased charge transfer resistance (Figure S8f, Supporting Information) also indicates that the morphology is destroyed at a certain level over 7000 cycles. Hence, there is a decrease in stability performance.

Figure 3g,h represents the electrochemical impedance spectroscopy (EIS) study of NMO‐4, NMO‐5, NMO‐6, and NMO‐7 h electrodes. The EIS study of all spectra was taken in 2m KOH electrolyte in the range between 100 kHz to 1 Hz. The Nyquist plot of NMO‐4, NMO‐5, NMO‐6, and NMO‐7 h electrodes were present in Figure 3g. The Figure S9 (Supporting Information) represents the R(C(RW)) equivalent circuit. It includes solution resistance (Rs), which accounts for the ionic conductivity of the electrolyte and resistances in the experimental setup. Next, the parallel combination of charge transfer resistance (Rct), capacitance (C), and Warburg impedance (W) models the processes at the electrode‐electrolyte interface. From the Nyquist plot the value of Rs were assessed for NMO‐4, NMO‐5, NMO‐6, and NMO‐7 h electrodes are 1.7, 1.5, 1.2, and 1.6 Ω cm^−2^ respectively. The minimum Rs represents the ionic conductivity of the electrolyte and the resistive contributions of the cell setup, influencing the efficiency of charge transport. The R_ct_ for NMO‐4, NMO‐5, NMO‐6, and NMO‐7 h electrode are 1.1, 1.9, 0.1, 1.2 Ω cm^−2^ respectively. The Rct reflects the resistance to Faradaic reactions at the electrode‐electrolyte interface, with lower 𝑅𝑐𝑡 values indicating faster charge transfer kinetics and improved power capability. The lower value of R_s_ and R_ct_ revealed that the NMO‐6 h sample shows a higher electrical conductivity. The W represents ion diffusion within the porous structure of the electrode, capturing the diffusion‐controlled behavior of the material. The R(C(RW)) equivalent circuit is well‐suited for describing the electrochemical behavior of NiMoO_4_ materials from the EIS data. These parameters together describe energy storage (C, W) and power capability (Rs, Rct), making this circuit suitable for analyzing and optimizing NiMoO_4_ for supercapacitor applications.^[^ ^38^ ^]^

It is important to note that the formation of microspheres reduces the diffusion path of electrolytic ions and improves charge transfer kinetics. Figure 3h shows the Bode plot of NMO‐4, NMO‐5, NMO‐6, and NMO‐7 h electrode samples. A phase angle (θ) closer to 45° or lower indicates the presence of Warburg impedance, which represents ion diffusion within the material. This is a typical feature of battery‐like behavior with significant charge storage due to bulk material reactions.^[^ ^39^ ^]^ As frequency increases, the phase angles for all samples approach zero. The NMO‐6 h at 160 °C shows enhanced electrochemical performance, with lower impedance and greater capacitive characteristics. Figure S10a–c (Supporting Information) shows the comparative electrochemical characteristics of bare nickel foam with NMO‐6 h sample including CV, GCD, and EIS study.

The overall supercapacitor study of the time series analyzed that the NMO‐6 h electrode at reaction temperature 160 °C exhibits maximum capacity than the other electrode and optimized the 6‐ h reaction time. Also, for further improvement have investigated the calcination temperature in the range of 350, 400, and 450 °C. After investigation of calcination effect, we never observed results better than 400 °C, which is previously studied in time effect. But the electrochemical results related to effect of calcination on NiMoO_4_ are mentioned in Figures S11 and S12 (Supporting Information). Mostly in the literature study the specific capacitance in F g^−1^ is calculated but for comparison of specific capacity in mAh g^−1^ Equation S2 (Supporting Information) is employed and calculated values are mentioned in Table 1.

Analyzing the capacitive and diffusion contributions to charge storage mechanisms in NiMoO_4_ electrodes is crucial for understanding their electrochemical performance. Equation (3) is the powers law, which is the relation between positive scan peak current (*i_p_*) and their corresponding scan rates (𝑣) from CV study.^[^ ^48^, ^49^ ^]^

(3) $$i_{p}=av^{b}$$

where, a and b are the constant parameters. The value of *b* = 0.5 then the material is said to be battery type, *b* = 1 then material is said to be capacitor type and the value of b is lies between 0.5 and 1 0.5 < *b* < 1 it is said to be supercapattery material. This means that the material shows both capacitor and battery type behavior from their electrochemical study.^[^ ^50^ ^]^ The linearity observed in the oxidation and reduction current peaks in Figure S13A (Supporting Information), with a correlation coefficient (R^2^) value of ≈0.999, confirms that the majority of the current is generated from a diffusion‐controlled electrochemical reaction. The optimized NiMoO_4_ electrode material exhibits b value of 0.76 for the cathodic process is depicted in Figure S13B (Supporting Information). The calculated b‐value indicates a hybrid charge storage mechanism in the material, involving both capacitive and diffusion‐controlled processes. In hybrid materials like NiMoO_4_, the capacitive contribution typically dominates at higher scan rates due to rapid charge storage at the surface of the material.^[^ ^49^ ^]^ This mixed behavior is beneficial as it allows for superior kinetics, meaning the material can effectively and quickly facilitate electrochemical reactions, which is advantageous for energy storage applications. Figure S13C (Supporting Information) shows that diffusion contribution is higher at lower scan rates (𝑣 = 5 mV s^−1^) while the capacitive contribution is higher and predominant at elevated scan rates (𝑣 = 100 mV s^−1^). NiMoO_4_ may have a highly mesoporous structure and efficient ion diffusion pathways, allowing diffusion‐controlled redox reactions to continue contributing significantly to charge storage even at high scan rates. However, Figure 3i shows that diffusion‐controlled processes remain significant even at higher scan rates. This phenomenon could be attributed to the unique structural properties of the material. Furthermore, the presence of slow faradaic reactions within the bulk of the material may also sustain diffusion control under rapid charge‐discharge conditions. This suggests that while the surface interactions (capacitive) occur quickly, the bulk material's contribution to the charge storage, via ion intercalation or diffusion‐controlled processes, remains substantial across a wide range of scan rates. This highlights the hybrid nature of the material and its ability to balance surface and bulk processes, making it suitable for energy storage applications requiring high capacity and stability.^[^ ^49^ ^]^ Figure 3i illustrates that the blue shaded area represents the diffusion‐controlled current, showing significant peaks indicating higher current at certain potentials, typical of redox processes involving mass transport. The red shaded area represents the capacitive controlled current, which is more uniform and closer to the baseline, indicating charge storage in the electric double layer.

### 2.2 Density Functional Theory Calculations of NiMoO4

We used XRD analysis of NiMoO_4_ to uncover the ion‐diffusion and transport mechanism. From XRD analysis it is found that structure shows that it is a crystallizes in the monoclinic C2/m space group with lattice parameters a = 9.54803 Å, b = 8.73855 Å, c = 7.62656 Å, and β = 113.9750°. The structure features two inequivalent sites each for Mo^6+^ and Ni^2+^ ions, both in 6‐coordinate geometry with oxygen atoms. The Mo─O bond distances range from 1.72–2.34 Å, while Ni─O bonds span 1.96–2.10 Å. Oxygen atoms occupy five distinct sites with varying coordination environments. Analysis of the structure reveals there are 30 polyhedra within the unit cell. To investigate the electronic properties of NiMoO_4_, we performed DFT calculations using the computational quantum mechanical modeling Quantum ESPRESSO code.^[^ ^51^, ^52^ ^]^ Our computational approach followed a four‐step procedure such as structural relaxation, self‐consistent field (SCF) calculation, non‐self‐consistent calculation (NSCF), DOS calculation, and band structure calculation. The SCF calculations were performed using the PBEsol exchange‐correlation functional with a plane‐wave cut off of 50 Ry for wavefunctions and 400 Ry for charge density and a Gaussian smearing of 0.02 Ry. We employed ultra‐soft pseudopotentials for Ni and norm‐conserving pseudopotentials for Mo and O atoms.^[^ ^53^, ^54^ ^]^ The calculations included spin polarization also to account for strong electron correlations in the partially filled d‐orbitals of Ni and Mo, which are not adequately described by standard DFT methods, we applied the DFT+U method. This approach helps to correct the self‐interaction error and improves the description of localized d‐states, which is crucial for accurately representing the electronic structure of transition metal molybdates like NiMoO_4,_^[^ ^55^ ^]^ we applied the DFT+U method with Hubbard U values of 4.38 eV for Mo and 6.2 eV for Ni close to the previous work.^[^ ^56^ ^]^ The Brillouin zone was sampled using a 4 × 4 × 5 Monkhorst‐Pack k‐point grid.

**Figure 4:**
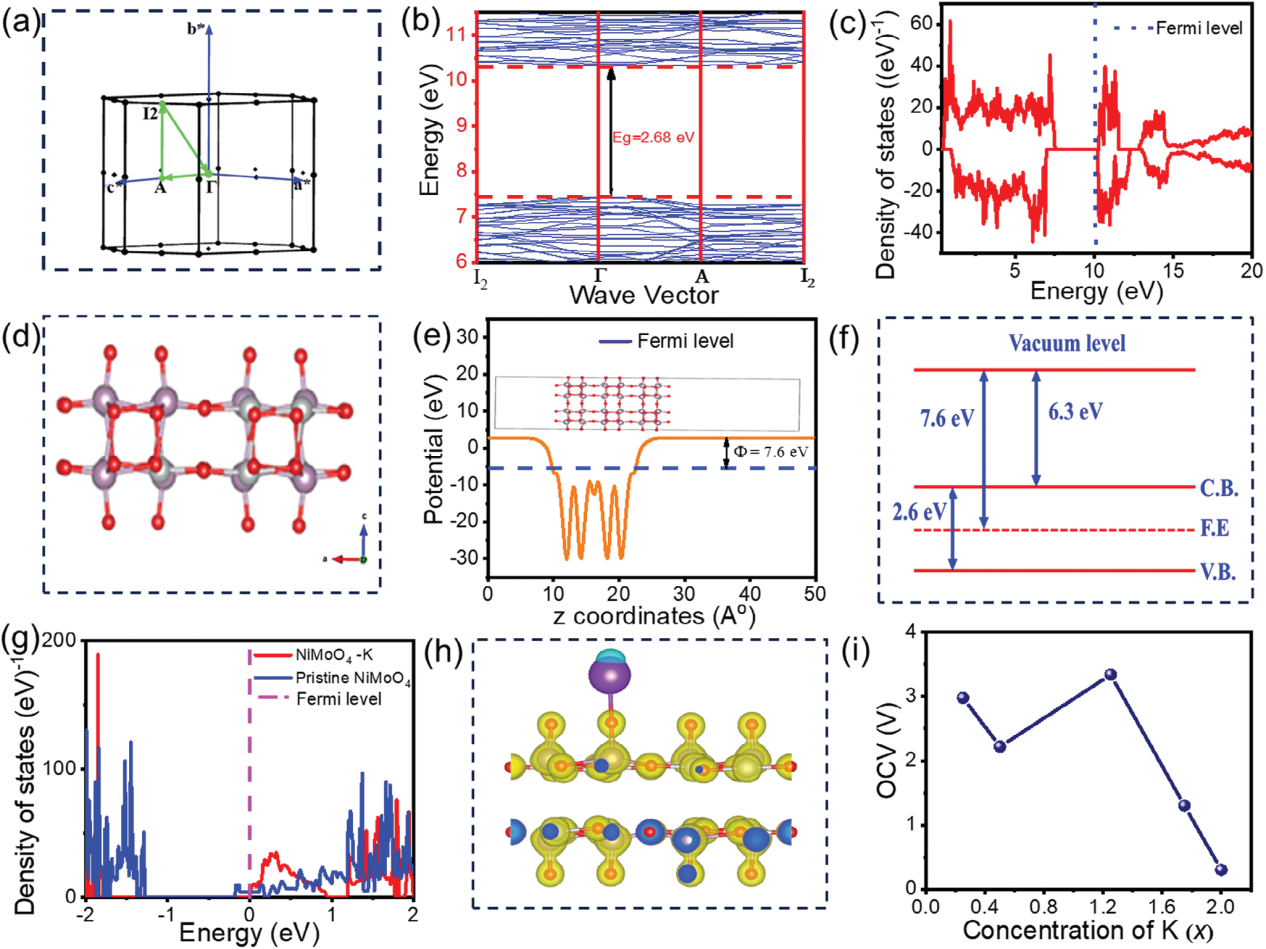
a) high symmetry k points b) band structure c) Density of states of bulk NiMoO_4_ d) crystal structure of pristine NiMoO_4_ for [110] plane e) work function f) energy level diagram g) Density of states of pristine NiMoO_4_ and NiMoO_4_‐K material h) iso‐surface plot for K adsorbed NiMoO_4_ material. i) Open circuit voltage Vs content of K atom in NiMoO_4_ material.

The energy bands are plotted along a high symmetry k point (I_2_, Γ, A, I_2_) as shown in **Figure** **4**a, with the energy scale referenced to the Fermi level (E_f_). The band structure of NiMoO_4_, as depicted in Figure 4b, exhibits a complex interplay of energy bands across the Brillouin zone. Notably, the bands are densely packed in the energy range from ≈6 to 11 eV, with a clear distinction between occupied and unoccupied states with the direct band gap along the gamma point is ≈6.68 eV. Below the E_f_, the bands are relatively flat, suggesting localized states with limited dispersion. This indicates that these states likely originate from the inner orbitals of the constituent atoms, which do not strongly hybridize. Above the E_f_, the bands become more dispersive, particularly around the high‐symmetry points in the k‐path, indicating the presence of delocalized states that result from the linear combination of atomic orbitals. These dispersive bands are characteristic of the conduction states in NiMoO_4_, where the overlapping orbitals contribute to electron mobility. The shape of the band structure changes with energy, with some bands showing significant curvature, suggesting strong orbital interactions and hybridization. This curvature in the bands is indicative of the varying degree of orbital overlap as the energy increases, with the transition metal d‐orbitals likely playing a significant role in the conduction band.

The calculated DOS for NiMoO_4_ reveals a complex electronic structure characteristic of transition metal molybdates. The DOS spans an energy range from 0 to 20 eV as shown in Figure 4c, with the E_f_ positioned at ≈10.2 eV. A notable feature is the presence of a small band gap of 2.68 eV just below the E_f_ which is close to the previous study,^[^ ^57^, ^58^ ^]^ where the DOS approaches zero for both spin orientations, indicating that NiMoO_4_ is likely a narrow‐gap semiconductor.^[^ ^59^ ^]^ The electronic structure exhibits distinct regions: below 8 eV, the DOS is characterized by pronounced peaks and valleys, corresponding to discrete energy levels in the valence bands between 8 and 10 eV, a region of minimal DOS signifies the band gap above 10 eV, the DOS increases again, representing the conduction bands. Notably, the spin‐polarized nature of the DOS, evident from the asymmetry between spin‐up and spin‐down states, particularly near the E_f_. This spin polarization becomes less pronounced at higher energies (>15 eV), where the DOS exhibits a smoother and more symmetric profile. The observed electronic structure, particularly the narrow band gap and spin polarization near the E_f_, is crucial for understanding the electrical behavior of NiMoO_4_ and its potential applications in energy storage.

The work function is essentially the energy barrier that must be overcome to move an electron from the surface of a solid material into the surrounding free space.^[^ ^60^, ^61^ ^]^ In our specific slab calculations, we investigated the work function for a 5‐layered slab oriented along the [110] plane, with a vacuum layer both above and below the structure measuring 10 and 30 Å in thickness respectively. The structure of pristine NiMoO_4_ [110] is shown in Figure 4d.

The energy barrier is visually represented in Figure 4e. The plot of atomic coordinates and the electrostatic potential is shown in k.

To ensure the accuracy of our calculations, we set the kinetic energy cut‐off for orbitals and the energy cut‐off for charge density expansion to 60 Ry and 600 Ry, respectively. These values were validated by confirming convergence in both the total energy and lattice structure parameters. For Brillouin zone integrations, we employed the gpaw code^[^ ^62^ ^]^ with a smearing parameter of 0.02 Ry. The k‐point mesh used for the slab model consisted of a 2 × 2 × 1 grid. According to work function calculation 7.1 eV energy is required to eject the electron. The calculated band gap energy is 2.6 eV. Figure 4f represents the visualization band structure from calculated parameters. The fermi energy to vacuum distance is 7.6 eV. Hence the conduction band to vacuum level having a distance of 6.3 V. For the development of band structure fermi energy level is placed exactly in between the center of the valence band and conduction band. Figure 4g represents the DOS of pristine NiMoO_4_ (blue line) and after K adsorption (red line). For pristine NiMoO_4_, the DOS shows a significant peak just below −1 eV, followed by a series of states up to 2 eV, indicating the presence of both valence and conduction states near the Fermi level. This implies that pristine NiMoO_4_ has accessible electronic states close to the Fermi level, suggesting its potential for charge storage and transfer. Upon K adsorption, the DOS undergoes notable changes, especially near the Fermi level. It indicates the emergence of new energy states in the vicinity of 0 eV, which can be attributed to the interaction between K^+^ atoms and the NiMoO_4_ structure. These additional states near the Fermi level indicate improved conductivity and enhanced electron mobility. This shift suggests that K^+^ adsorption modifies the electronic structure, potentially enhancing the material's charge transfer capabilities. The DOS illustrates that the pristine NiMoO_4_ already exhibits a suitable electronic structure for energy storage. However, the adsorption of K^+^ significantly alters its electronic properties, introducing new states near the Fermi level, likely enhancing its performance as a cathode material in supercapacitors by improving its charge storage and conductivity. The charge density difference plot shown in Figure 4h also shows the charge conductivity, the yellow color shows the electron accumulation and the cyan color shows the electron depletion.

In supercapacitors and batteries, the performance of cathode materials is essential, as they play a critical role alongside electrodes. The cathode should exhibit a high open‐circuit voltage (OCV) to generate a strong electric potential. To improve output voltage and overall efficiency, the positive electrode material should ideally meet several key criteria, it should have a stable electrochemical potential, retain structural integrity even after full adsorption of metal ions during the charging process, and possess a high specific capacity.^[^ ^63^, ^64^ ^]^ As the concentration of K^+^ metal ions increases, the average adsorption energy gradually decreases due to interactions within the adsorbed layers. The OCV of K atoms is calculated using Equation (4) and it is shown in Figure 4i,

(4) $$OCV=-\frac{E_{ad}}{e}$$

where E_ad_ is the average adsorption energy of a metal atom and e is the charge of an electron. The following Equation (5) is used to calculated the specific capacity for K^+^ ions storage,^[^ ^27^ ^]^

(5) $$C=\frac{xnF}{M+nM_{m}}$$

where, *M* is the molecular weight of monolayers, *M_m_* is the molecular mass of the metal atom (K) and *F* is Faraday constant (26 801 mAh mol^−1^), *x* is the chemical stoichiometry of the K atom and *n* is the valency of potassium ion.

To determine the appropriate concentration values (*x*), K atoms were added incrementally, and the adsorption energy was recalculated after each addition. This approach allowed us to observe how increasing K concentration affects the system. The concentration values are set at intervals (e.g., 0.25, 0.5, 1.25, 1.75) based on the number of K atoms added relative to the base structure. As *x* approaches 2, the increased concentration of K leads to repulsion between metal ions, causing the K adsorption energy to decrease and concentration was reached to its maximum limit. Some of the calculations showed the convergence, indicating a more stable adsorption configuration as the concentration increased reflecting favorable conditions for adsorption. The specific capacity was calculated as 203 mAh g^−1^ when the K metal atom concentrations reached the condition where *x* = 2.

**Figure 5:**
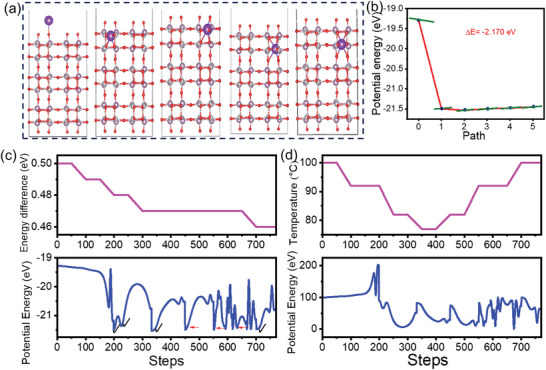
a) Diffusion path of potassium ion for NiMoO_4_ material, atomic configuration for local minima b) NEB path for diffusion of potassium ion on NiMoO_4_ [110] plane. c) energy difference and potential energy versus simulation steps d) temperature and potential energy versus simulation steps.

To study the interaction between KOH and the NiMoO_4_ [110] surface was investigated using constrained minima hopping (CMH) global optimization implemented in the Atomic Simulation Environment (ASE).^[^ ^65^, ^66^ ^]^ The initial NiMoO_4_ structure with an adsorbed K atom was placed on the top sit of NiMoO_4_ [110] plane with three layers. The last layer of the structure was fixed and the upper two layers with K^+^ atoms were kept free to move. Interatomic interactions were modelled using a Lennard–Jones potential.^[^ ^67^ ^]^ The CMH algorithm was initialized with E_diff_ = 0.5 and T_0_ = 100 K, running for 10 steps to explore the potential energy surface and identify low‐energy configurations of K^+^ on NiMoO_4_ for [110] plane. This approach facilitates the study of K^+^ diffusion pathways and potential KOH reaction sites on the NiMoO_4_ surface. The results of the CMH calculation are presented in **Figure** **5**a where all figures of Figure 5a show the atomic configuration where the minimum was accepted during the MD simulation. Figure 5b illustrates the minimum energy pathway for the diffusion of the K^+^ atom on the NiMoO_4_ [110] surface during the three key steps identified in the CMH calculation. The plot shows the energy profile as a function of the reaction coordinate. The initial and final states of the K^+^ atom, represented by the blue points on the curve, correspond to the local minima identified during the CMH steps. The energy difference (ΔE) between the initial and final states is ≈−2.17 eV, indicating a significant energy drop as the K^+^ atom moves from the higher‐energy state to a more stable, lower‐energy configuration. The smooth black curve connecting these states represents the calculated minimum energy pathway, while the green lines at the endpoints and intermediate points show the tangent to the potential energy surface at those points. This pathway provides insight into the energy barrier for K^+^ atom diffusion across the NiMoO_4_ surface and highlights the stable adsorption sites. The evolution of potential energy (E_pot_) and E_diff_ which is a self‐adjusting parameter are plotted against the simulation steps is shown in Figure 5c. The temperature profile (K) varied slightly ≈100 K Figure 5d, allowing the algorithm to explore different configurations by slightly altering the kinetic energy of the system. The E_diff_ plot shows a gradual decrease in energy differences from 0.5 eV to ≈0.46 eV, indicating the identification of progressively lower‐energy configurations. The E_pot_ plot demonstrates the systems search for the most stable configurations. A checkmark signifies that the local minimum was accepted, while red arrows point out instances where it was rejected. The blue line connecting the steps represents the potential energy during the molecular dynamics (MD) phase of each step. The potential energy oscillating between higher‐energy states and converging toward lower‐energy minima ≈−21.5 eV. This indicates the successful identification of several low‐energy configurations, which are likely to be favorable sites for K^+^ atom adsorption on the NiMoO_4_ [110] plane surface. The significant energy drops observed at certain steps suggest the discovery of new minima, highlighting potential diffusion pathways of K^+^ atoms across the surface. These findings provide valuable insights into the interaction mechanisms between KOH and NiMoO_4_, which could be critical for optimizing the surfaces electrochemical properties.

### 2.3 Fabrication and Performance Evaluation of NiMoO_4_//AC Hybrid Supercapacitor

**Figure 6:**
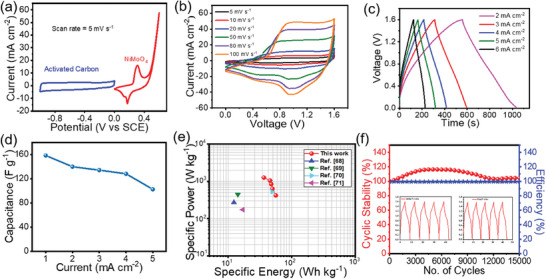
a) CV curves of the positive and negative electrodes measured at a constant scan rate of 5 mVs^−1^. b) CV curves of the HSC device recorded at various scan rates c) GCD curves of the HSC device recorded at different current densities (2–6 mA cm^−2^), respectively. d) Variation of specific capacitance as a function of the current density of HSC device. e) Ragone plot of HSC device at different current densities (2–6 mA cm^−2^). f) cyclic stability and coulombic efficiency of an aqueous symmetric device over 15 000 continuous GCD cycles at 100 mA cm^−2^. Inset initial and final 5 cycles.

The assembled HSC NiMoO_4_//AC device was developed using NiMoO_4_ electrode as anode and AC as a cathode in an aqueous 2m KOH electrolyte. The purpose of the study was to investigate how well the HSC device, and the AC electrode performed. First, we have tested AC electrode using CV and GCD study to evaluate its performance. The Equation S4 (Supporting Information) was used to predict the optimum mass of the negative electrode material (≈2 mg cm^−2^), which was occupied to balance the charges on both electrodes of the HSC device. Parameters such as specific capacitance, specific energy, specific power, and capacity retention are measured. **Figure** **6**a shows the CV curves of NiMoO_4_ microspheres and AC electrodes and measured their potential voltage range between 0.0 V–0.6 V and −1.0 V–0.0 V at scan rate of 5 mV s^−1^, respectively. This figure suggested that the working potential window was 1.6 V, which is the total of the potential windows of the positive and negative electrodes, making it suitable for HSC device. The HSC device was tested for CV and GCD within a potential range of 0–1.8 V to optimize the potential window (Figure S14a,b, Supporting Information). From the analysis of the CV curves at scan rate 5 mV s^−1^, it was observed that the potential range reached up to 1.6 V. Beyond this point, the CV scan showed an oxygen evolution hump during the positive scan. Also, from GCD analysis at a current density of 7 mA cm^−2^, the GCD charging curve has not reached a potential of 1.7 V. Therefore, we selected the voltage window of 0–1.6 V as it is more suitable for investigating the electrochemical performance of the HSC NiMoO_4_//AC device. The CV curves of NiMoO_4_//AC device at a scan rate of 5–100 mV s^−1^ is depicted in Figure 6b. The current response increases with the scan rate, which indicates the electrochemical activity of the HSC device increases with faster potential sweeps. The CV curves are suggesting reversible redox processes, particularly the presence of peaks, which indicates both capacitive and battery type redox reactions occurring within the device. Figure 6c shows the GCD curves at different current densities of 2–6 mA cm^−2^ within the potential range 0–1.6 V. The ASC device delivered enormous specific capacitance of 158.3, 139.7, 134.1, 128.1, and 102.3 F g^−1^ at current densities of 2, 3, 4, 5, and 6 mA cm^−2^, respectively. The symmetric charge‐discharge nature of the HSC device maintains its initial capacitance of 65% up to at current density of 6 mA cm^−2^ is shown in Figure 6d. The HSC NiMoO_4_//Ac device displayed a higher specific energy of 56.3 Wh kg^−1^ at a specific power of 421 W kg^−1^ from Equations (S5) and (S6) (Supporting Information). Outstandingly, the HSC device exhibited a specific energy of 36.4 Wh kg^−1^ at a higher specific power of 1263.2 W kg^−1^ is shown in Ragone plot Figure 6e compared with the previously reported data.^[^ ^68^, ^69^, ^70^, ^71^ ^]^ The fabricated HSC device delivered an enormous cyclic performance of 104.46% and coulombic efficiency of 100% after 15 000 cycles at a higher current density of 80 mA cm^−2^ is shown in Figure 6f. During the initial cycles, the performance improvement is primarily due to the activation of the NiMoO_4_ electrode. This activation involves improved wettability of the NiMoO_4_ surface by the electrolyte and better penetration of the electrolyte into its porous structure, enabling more effective utilization of the active sites. Simultaneously, structural rearrangements in the NiMoO_4_ nanostructures during cycling can expose additional active sites for faradaic redox reactions, enhancing the electrochemical activity. On the negative side, the activated carbon provides stable double‐layer capacitive behavior, complementing the redox activity of NiMoO_4_. After reaching a peak in capacitance retention, typically ≈6000 cycles, a gradual decline occurs due to several degradation mechanisms. For NiMoO_4_, repeated volume changes during faradaic redox reactions can lead to structural instability, including cracking or pulverization, resulting in the loss of active sites. Electrolyte decomposition over extended cycling may also form resistive films on the NiMoO_4_ surface, reducing ionic accessibility.^[^ ^72^ ^]^ Inset of Figure 6f shows the initial and final 5 charging discharging cycles of NiMoO_4_//AC device at current density of 80 mA cm^−2^. Figure S15 (Supporting Information) shows the structural, morphological, and electrochemical analysis of positive electrode (NiMoO_4_) of the HSC device after 15 000 cycles. The XRD pattern (Figure S15a, Supporting Information) shows that no substantial changes in the electrode sample even after 15 000 cycles. Figure S15b,c (Supporting Information) shows SEM images of the positive electrode (NiMoO_4_) of the HSC device. Even after stability testing, the NiMoO_4_ electrode retains its spherical structure, with nanorods still visible. The morphology appears largely intact, suggesting excellent structural stability during prolonged cycling. The comparative CV, GCD, and EIS characteristics of the NiMoO_4_//AC HSC device before and after cyclic stability testing is depicted in Figure S15d–f (Supporting Information). This study shows that the HSC device delivered excellent cyclic performance over 15 000 GCD cycles. The CV and GCD graphs indicate an improvement in the HSC devices performance after 15000 GCD cycles (Figure S15d,e, Supporting Information). From the Nyquist plot the values of Rs were assessed for before and after stability electrode are 1.2 and 1.3 Ω cm^−2^ respectively. The minimum Rs represents the ionic conductivity of the electrolyte and the resistive contributions of the cell setup, influencing the efficiency of charge transport after stability. The Rct for before and after stability electrodes are 0.3 and 0.5 Ω cm^−2^ respectively. In the Nyquist plot study, the reduction in charge transfer resistance after cycling indicates improved electrochemical performance and stability of the device. The inset of Figure S15c (Supporting Information) depicts the R(C(RW)) comparable circuit. It comprises Rs, which accounts for the electrolyte's ionic conductivity as well as experimental setup resistances. Also, the electrode‐electrolyte interface processes are modeled using a parallel combination of Rct, C, and W. The stable Warburg impedance suggests that the device maintains good ion diffusion properties even after extensive cycling, highlighting its suitability for long‐term electrochemical applications. Lastly, we have also tested the symmetric device of NiMoO_4_//NiMoO_4_ device in 2M KOH, which delivered a less performance as compared to HSC device (Figures S16 and S17, Supporting Information).

## 3 Conclusions

This study highlights the potential of NiMoO_4_ as a promising material for energy storage applications, particularly in hybrid supercapacitors. NiMoO_4_ microspheres, deposited on nickel foam via a hydrothermal method with optimized reaction conditions, demonstrated superior electrochemical performance, delivering a specific capacity of 168.9 mAh g^−1^ at 1 A g^−1^ and retaining 80% capacity over 7000 charge‐discharge cycles with 98.3% coulombic efficiency. The theoretical insights obtained through DFT provided a deeper understanding of the material's electrochemical behavior. DFT calculations revealed a for adsorption of K^+^ ions for the [110] plane of NiMoO_4_ and confirmed a theoretical specific capacity of 203 mAh g^−1^, make parallel closely with experimental results. Potassium ion diffusion pathways were identified using potential energy landscape modeling. Additionally, the HSC (NiMoO_4_//AC) demonstrated excellent performance, delivering a specific energy of 56.3 Wh kg^−1^ at a specific power of 421 W kg^−1^, with slight capacity loss after 15 000 cycles. This underscores the robustness and reliability of NiMoO_4_ as a battery‐type electrode material. The findings establish a strong correlation between the structural, theoretical, and electrochemical properties of NiMoO_4_, offering valuable insights into its potential for energy storage applications. The unique properties of NiMoO_4_ make it an excellent candidate for next‐generation energy storage devices. Its versatility in HSC configurations and compatibility with scalable, cost‐effective synthesis methods position it well for integration into renewable energy systems, electric vehicles, and grid‐scale energy storage applications. Furthermore, the insights provided by this study open avenues for exploring other multivalent ion systems, such as magnesium or zinc ions, which could further enhance energy density and cycling stability. These advancements would pave the way for NiMoO_4_ based devices to play a critical role in addressing global energy demands and transitioning toward sustainable energy solutions.

## 4 Experimental Section

### Synthesis of NiMoO_4_ Microflowers

**Figure 7:**
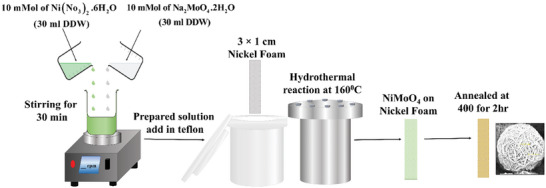
Schematic illustration of the NiMoO_4_ microsphere synthesis process.

Through simple hydrothermal treatment, the NiMoO_4_ thin films were created by controlling the calcination temperature and reaction time. In actual synthesis, 10 mM Ni (NO_3_)_2_.6H_2_O were dissolved in 30mL Double Distilled water (DDW) and 10 mm Na_2_MoO_4_.2H_2_O were dissolved in 30mL DDW in separate glass beakers and stirred for 30 min each. Then prepared final solution mixed with each other and the solution stirred for 30 min. The mixed solution and precleaned nickel foam used as substrates were ultrasonically cleaned with 5% HCl, ethanol, and DDW in a sequential manner and shifted into Teflon liner. Subsequently, the solution was then placed in a stainless‐steel autoclave, sealed, and heated in a hot air oven at 160 °C for 4, 5, 6, and 7 h, respectively. Upon completion of the reaction times, the NiMoO_4_ coated nickel foam substrates were rinsed 2–3 times with DDW and left to dry overnight at 60 °C. Finally, all dried NiMoO_4_ coated nickel foams were calcined at 400 °C for 2 h in an air atmosphere. The prepared electrode material was synthesized, calcinated, and abbreviated as NMO‐4, NMO‐5, NMO‐6, and NMO‐7 h, respectively, and concluded as NMO‐6 h is a good, optimized electrode material. The reaction time as 6 h was revealed good results, and then NiMoO_4_ electrode material was synthesized and calcinated for different temp at 350 °C, 400 °C, and 450 °C for 2 h, respectively by keeping other reaction parameters constant. Based on calcination temperature, the NiMoO_4_ thin films were named as NMO‐350, NMO‐400, and NMO‐450, respectively. From this series, the film calcined at 400 °C was identified as optimal. The deposited mass was determined using the weight difference method, yielding 1.2 mg across all variations of time and calcination temperature. The Schematic illustration is shown in **Figure** **7**. Also, the reaction growth mechanism was explained using Equations (S7–S9) (Supporting Information).

### Electrochemical Measurements

The electrochemical properties were investigated by the cyclic voltammetry, galvanostatic charge‐discharge study, and electrochemical impedance spectroscopy of NiMoO_4_ materials in 2 m KOH electrolyte. In the electrochemical measurements, the directly deposited NiMoO_4_ was used as a current collector or working electrode in three electrode system. While, saturated calomel and platinum (Pt) wire were used as the reference and counter electrodes, respectively. A Biologic Sp‐300 electrochemical workstation was used to perform all electrochemical properties. A hybrid SC device was assembled using directly deposited NiMoO_4_ microspheres on nickel foam as the positive electrode and AC as the negative electrode in an aqueous 2 m KOH electrolyte. The negative electrode was prepared by using AC as active material, PVDF, and carbon P black as a binder in a mass ratio of 80:10:10 with NMP was used as a solvent and all were crushed in agate mortar. The prepared slurry of the material was coated on precleaned 1×1 cm^2^ nickel foam.

## Conflict of Interest

The authors declare no conflict of interest.

## Supporting information



Supporting Information

## Data Availability

The data that support the findings of this study are available from the corresponding author upon reasonable request.
