# Supplementary material for: Theoretical Specific Capacity and Metal Ion Diffusion Pathway of NiMoO4 Microspheres for Hybrid Supercapacitors
Source: Small. 2025 Feb 24;21(13):2500080. doi: 10.1002/smll.202500080 (PMC11962703; doi:10.1002/smll.202500080)
Supplement: Supplementary file 1 — Supporting Information [file SMLL-21-2500080-s001.docx]

**Theoretical Specific Capacity and Metal Ion Diffusion Pathway of NiMoO_4_ Microspheres for Hybrid Supercapacitors**

Digambar S. Sawant^1,2^, Sandesh V. Gaikwad^2^, Akash V. Fulari^3^, Mani Govindasamy^4^, Shrinivas B. Kulkarni^1*^, Deepak P. Dubal^5*^, Gaurav M. Lohar^2*^

^1^Department of Physics, The Institute of Science, Dr. Homi Bhabha State University, Madam Cama Road, Mumbai 400032, India

^2^Department of Physics, Lal Bahadur Shastri College of Arts, Science and Commerce, Satara 415002.

^3^Symbiosis Centre for Nanoscience and Nanotechnology, Symbiosis International (Deemed University), Pune, India

^4^Research Center for Intelligence Medical Devices, Ming Chi University of Technology, New Taipei City 243303, Taiwan

^5^School of Chemistry & Physics, Centre for Material Science, Queensland University of Technology, Brisbane, QLD 4000, Australia

**Charge-discharge mechanism**

To assess electrode reversibility, we examined the CV curve at scan rates from 5-100 mV s^-1^ and the GCD curve at current densities ranging from 1-5 A g^-1^. The CV analysis revealed that, as the scan rate increased, the oxidation and reduction peaks shifted toward more positive and negative potentials, respectively, with peak current values rising progressively from lower to higher scan rates. These curves demonstrate that NiMoO_4_ has high reversibility characteristics and shows high capacitance. The GCD curves of the NiMoO_4_ microspheres electrode shows the discharge time for all curves are symmetric. The faradaic redox reactions can be described by the following Eq^n^ (S1) ^[1]^.

$$\left.\left\{\begin{matrix} NiMoO_{4}\leftrightarrow Ni^{2+}+MoO_{_{4}}^{2-} \\ Ni^{2+}+2OH^{-}\leftrightarrow Ni\left(OH\right)_{2} \\ Ni\left(OH\right)_{2}+OH^{-}\leftrightarrow NiOOH+e^{-} \end{matrix}\right.\right\}\text{ }.....(S1)$$

**Electrochemical measurements**

**(a) Three Electrode System:**

The electrochemical properties were investigated by the cyclic voltammetry (CV), galvanostatic charge-discharge (GCD) study, and electrochemical impedance spectroscopy (EIS) of NiMoO_4_ materials in 2 M KOH electrolyte. In the electrochemical measurements, the directly deposited NiMoO_4_ were used as current collector or working electrode in three electrode system. While, saturated calomel and platinum (Pt) wire were used as the reference and counter electrodes, respectively. A Biologic Sp-300 electrochemical workstation was used to perform all electrochemical properties. The specific capacity in mAh g^-1^ and of the NiMoO_4_ nickel foam electrode were calculated by using the following Eq^n^ (S2) from the GCD study ^[2]^.

$$\text{Specific capacity (mAh g}^{-1}\text{) = }\frac{I\times\Delta t}{3.6\times m}\text{ ......(S2)}$$

Where *I* is applying current at charge discharge process (mA), *Δt* is discharging time, m is the actual mass of material are present on working electrode (mg). All electrochemistry has been done in 2M KOH electrolyte.

The specific capacitance (F g^-1^) value in terms of specific capacity (mAh g^-1^) is given by in Eq^n^ (S3)^[3]^

$$\text{Specific Capacitance=}\frac{Specific\text{ capacity (mAh g}^{-1})\times3.6}{\Delta v}\text{ (S3)}$$

**(b) Fabrication of Hybrid Supercapacitor:**

HSC device is developed by using directly deposited binder free NiMoO_4_ microspheres optimized nickel foam sample as positive electrode and activated carbon (AC) as negative electrode material in aqueous 2M KOH electrolyte. The negative electrode was prepared by using AC as active material, PVDF and carbon P black as binder in mass ratio of 80:10:10 with NMP was used as solvent and all are crushing in agate mortar. The prepared slurry of the material was coated on precleaned 1×1 cm^2^ nickel foam. The mass of the negative electrode was calculated from the charge balanced theory based Eq^n^ (S4). The specific energy and specific power of the HSC were calculated by using following Eq^n^ (S5 & S6) ^[4,5]^.

$$\begin{matrix} \frac{m^{+}}{m^{-}}=\frac{Sc^{-}\times\Delta V^{-}}{Sc^{+}\times\Delta V^{+}}\text{ .....(S4)} \\ E_{S}=\frac{1}{2}S_{c}\Delta V^{2}\text{ .....(S5)} \\ P_{S}=\frac{E_{S}}{\Delta t}\text{ .....(S6)} \end{matrix}$$

Where, positive and negative signs indicate the anode and cathode, respectively. *ΔV* is voltage window of GCD study, Sc is specific capacitance in F g^-1^ from GCD study, E_S_ is the specific energy in Wh kg^-1^ and P_S_ is specific power in W kg^-1^.

**XRD analysis:**

The diffraction peaks appeared at 14.89º, 25.30º, 28.22º, 43.97º, 49.19º, 57.85º, 59.89º, 63.58º, 75.71º are attributed to crystal planes of (1 1 0), (-2 0 2), (2 2 0), (3 3 0), (0 4 2), (-5 3 2), (4 4 0), (-6 2 3), (-6 2 5) respectively.

**
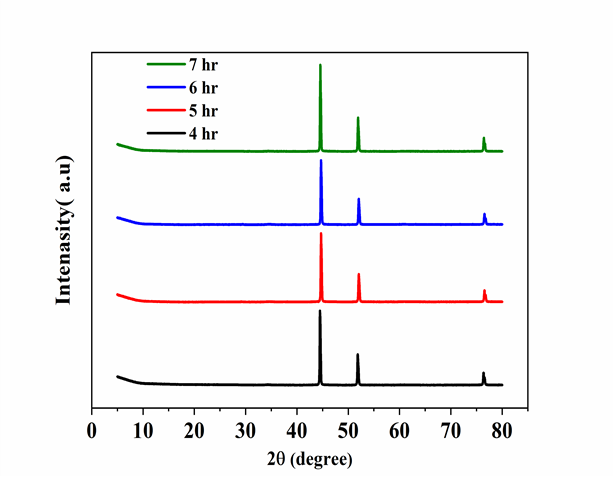
**

**Fig. S1** XRD of NMO-4hr, NMO-5hr, NMO-6hr and NMO-7hr time series samples

**
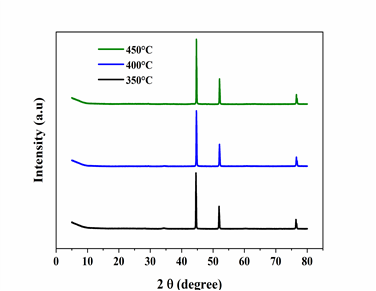
**

**Fig. S2** XRD of NMO-350, NMO-400, NMO-450 calcinated series samples

**
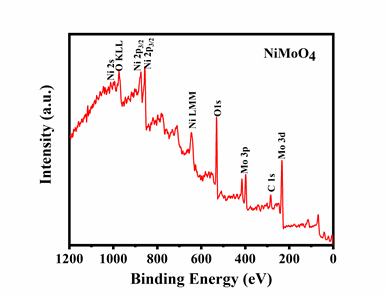
**

**Fig. S3** Total wide scan XPS survey spectrum of optimized NMO-6hr material


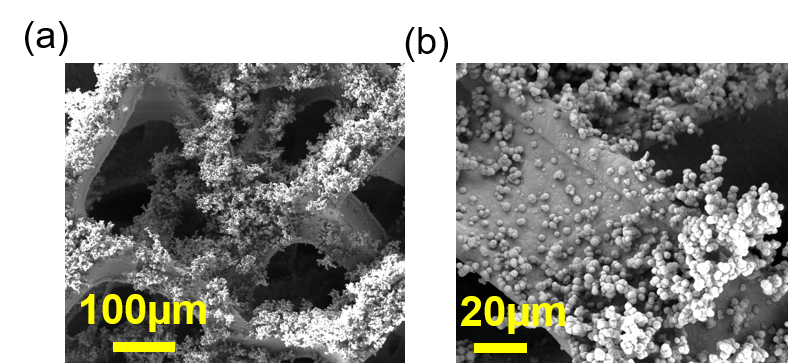


**Fig. S4** SEM images of NiMoO_4_ microspheres on nickel foam at lower magnification**.**

**SEM of Calcination Series:**

The morphology-based electrochemical analysis suggested that the NMO-6hr sample, synthesized at a reaction temperature of 160 ℃, was the optimized sample and was used for further calcination temperature studies. **Fig. S5(a-c)** shows the FESEM images of the calcination series for NMO-350℃. When the calcination temperature was reduced to 350℃, the size of the microspheres increased to approximately 4 µm. The optimized sample, NMO-400℃, was calcined at 400℃ **(Fig. S5(d-f)).** Further NMO-6hr sample calcinated at 400℃ results in microspheres with a diameter of approximately 2.6 μm. This size of microspheres is very low in size than the other calcination series samples. Finally, the NiMoO₄ optimized sample was calcined at a higher temperature of 450℃ and is referred to as NMO-450℃ **(Fig.** **S5(g-i)).** At this higher calcination temperature, the microspheres further increased in size to around 7.3 µm. FE-SEM analysis confirms that the NMO-400℃ sample developed well-defined microspheres with agglomerated nanorods. The change in microsphere size with varying calcination temperatures is primarily due to the effects of temperature on the crystallization, grain growth, and sintering processes. As the calcination temperature increases, the energy provided to the system promotes the mobility of ions within the NiMoO_4_ structure, leading to enhanced grain growth and the formation of larger microspheres ^[6]^. This temperature-dependent behavior underscores the importance of optimizing calcination conditions to control the morphology and achieve desired electrochemical properties.


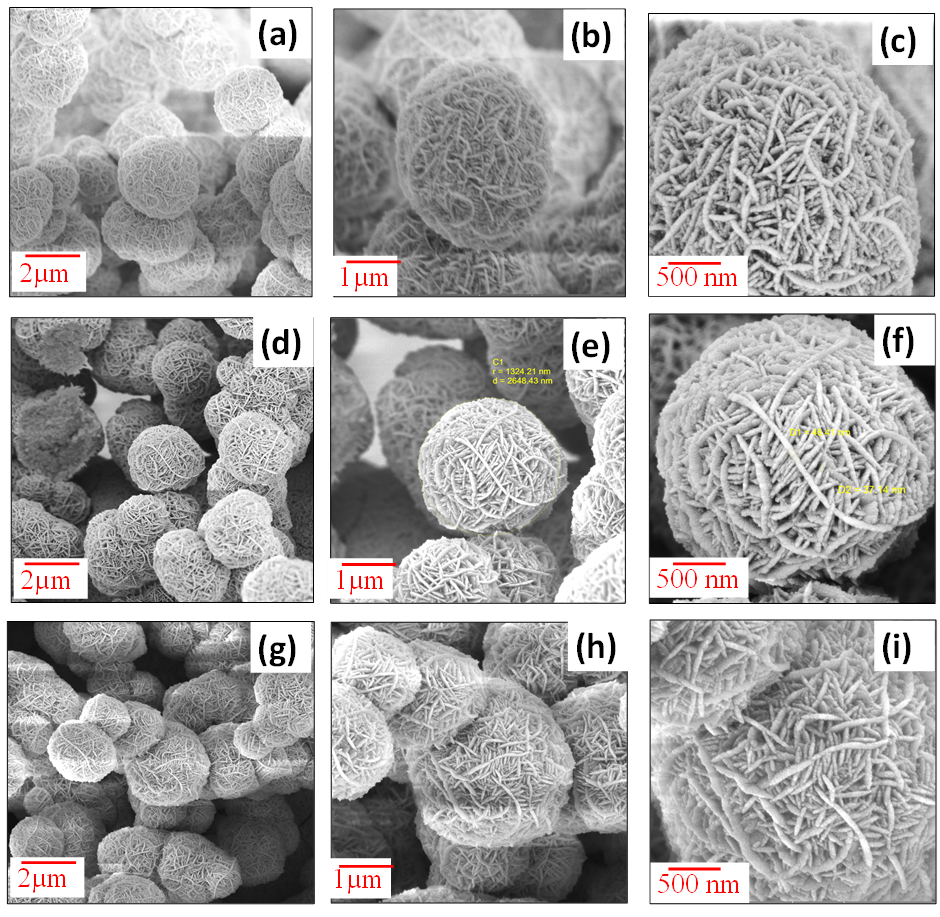


**Fig. S5** (a-c) SEM images of NMO-350℃, (d-f) NMO-400℃, (g-i) NMO-450℃ samples, respectively.

**
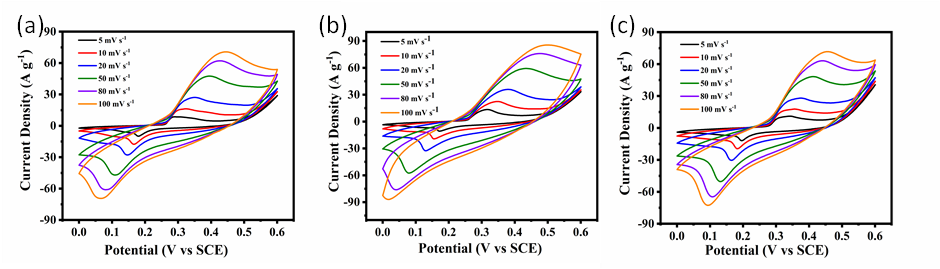
**

**Fig. S6** Cyclic voltammetry of (a) NMO-4hr, (b) NMO-5hr, and (c) NMO-7hr time series samples.

**Fig. S7** Galvanostatic charge discharge study of (A) NMO-4hr, (B) NMO-5hr, and (C) NMO-7hr time series samples.


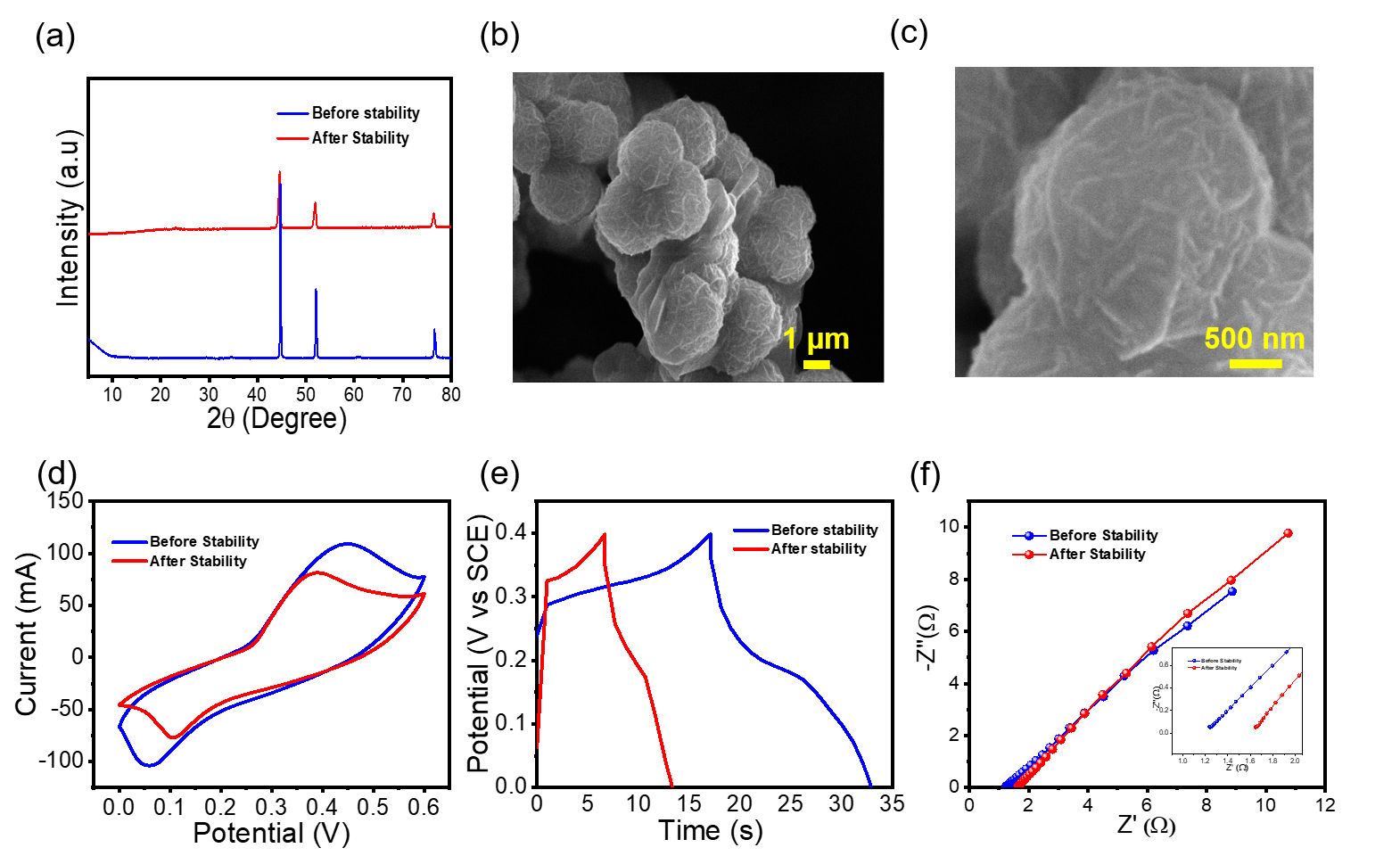


**Fig. S8** (a) Comparative XRD of the before and after stability NiMoO_4_ samples. (b-c) SEM images of after stability sample of NiMoO_4_. (d-f) Comparative CV, GCD and EIS study of NMO-6hr sample before and after stability.

**Fig. S9** Equivalent Circuit Diagram


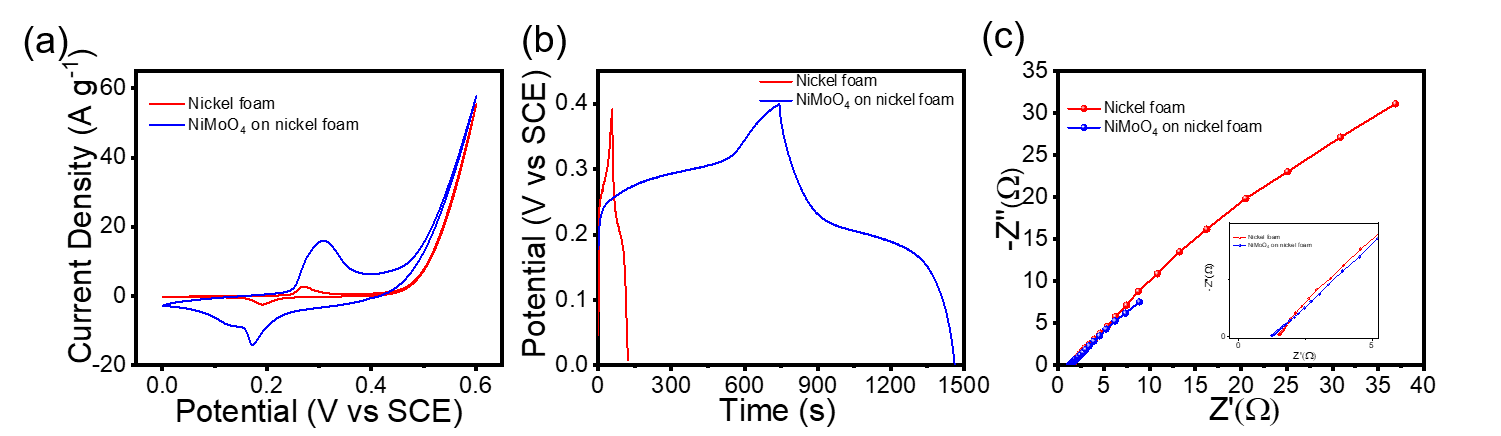


**Fig. S10** (a-c) CV, GCD and EIS of nickel foam compared with NMo-6hr Sample.

**Electrochemistry of Calcination series of NiMoO_4_**

**Fig. S11(a & b)** depicts the overall electrochemical study of temperature series. **Fig. S11(a)** **s**hows the cyclic voltammetry (CV) curves of NMO-350℃, NMO-400℃ and NMO-450℃ electrode at minimum scan rate of 5 mV s^-1^. **Fig. S11(b)** represents the galvanostatic charge discharge (GCD) curves of NMO-350℃, NMO-400℃ and NMO-450℃ electrode at minimum current density of 1 A g^-1^. **Fig. S11(c)** shows the specific capacity value of NMO-350℃, NMO-400℃ and NMO-450℃ at different current densities (1-5 A g^-1^). The maximum capacity and were calculated from GCD curves for NMO-350℃, NMO-400℃ and NMO-450℃ electrode are 115.74, 168.98 and 74.53 mA h g^-1^ from the **Eq^n^ (S2)** respectively. **Fig. S12(a, b)** represents the CV curves ofNMO-350℃ and NMO-450℃ at different scan rate (5-100 mV s^-1^). **Fig. S12(c, d)** represents the GCD curves of NMO-350℃ and NMO-450℃ at different current density (1- 5 A g^-1^). **Fig. S11(d-f)** represents the EIS study of NMO-350℃, NMO-400℃ and NMO-450℃ electrode. The EIS study of all spectra was carried out in 2M KOH electrolyte in the range between 1 Hz to 100 kHz. The Nyquist plot of NMO-350℃, NMO-400℃ and NMO-450℃ electrodes were present in **Fig. S11(d).** **Fig. S11(e)** represents the equivalent circuit. From the Nyquist plot the value of R_s_ were assessed forNMO-350℃, NMO-400℃ and NMO-450℃ electrode are 1.58, 0.1951, 1.981 Ω cm^-2^ respectively. The R_ct_ for NMO-350℃, NMO-400℃ and NMO-450℃ electrode are 0.5526, 0.01 and 0.2302 Ω cm^-2^respectively. The lower value of R_s_ and R_ct_ revealed that the NMO-6hr sample shows a higher electrical conductivity. **Fig. S11(f)** shows the Bode plot of NMO-350℃, NMO-400℃ and NMO-450℃ electrode sample. The phase angle(θ) is most negative at low frequencies for the 400°C sample, suggesting more significant capacitive behavior, while the phase angles for all samples converge toward zero at higher frequencies. Overall, the NiMoO_4_ sample annealed at 400°C demonstrates superior electrochemical performance, characterized by lower impedance and higher capacitive behavior, making it a more suitable candidate for applications such as supercapacitors.

**Fig. S11** (a) The comparative CV curves of NMO-350, NMO-400 and NMO-450 electrodes at 5 mV s^-1^ scan rate. (b) The comparative GCD curves of NMO-350, NMO-400 and NMO-450 electrodes at current density of 1 A g^-1^ (c) Variation of specific capacity as a function of the current density of NMO-350, NMO-400 and NMO-450 electrodes. (d) Nyquist plots of electrodes NMO-350, NMO-400 and NMO-450.Inset Fig. equivalent circuit diagram. (e) (f) Bode plot of electrodes NMO-350, NMO-400 and NMO-450 respectively.

**Fig. S12** Cyclic voltammetry of (a) NMO-350, (b) NMO-450 calcinated series samples. Galvanostatic charge discharge study of (c) NMO-350, (d) NMO-450 calcinated series samples.

**Fig. S13** (a) Peak currents with respect to the square roots of the different scan rates, (b) log of peak currents with respect to the log of scan rates. (c) Capacitive and diffusion-controlled contribution CV graph of NMO-6hr sample.

**Table S1. Specific capacity of the electrodes with discharging time.**

| Electrode Sample | Discharging time (s) | Specific capacity (mAh g^-1^) at 1 A g^-1^ |
| --- | --- | --- |
| NMO-4hr | 466 | 107.87 |
| NMO-5hr | 607 | 140.50 |
| NMO-6hr | 730 | 168.98 |
| NMO-7hr | 575 | 133.10 |
| NMO-350 | 500 | 115.74 |
| NMO-400 | 730 | 168.98 |
| NMO-450 | 322 | 74.53 |

**Fig. S14** (a) CV and (b) GCD of HSC device within the potential window of 0–1.8 V.


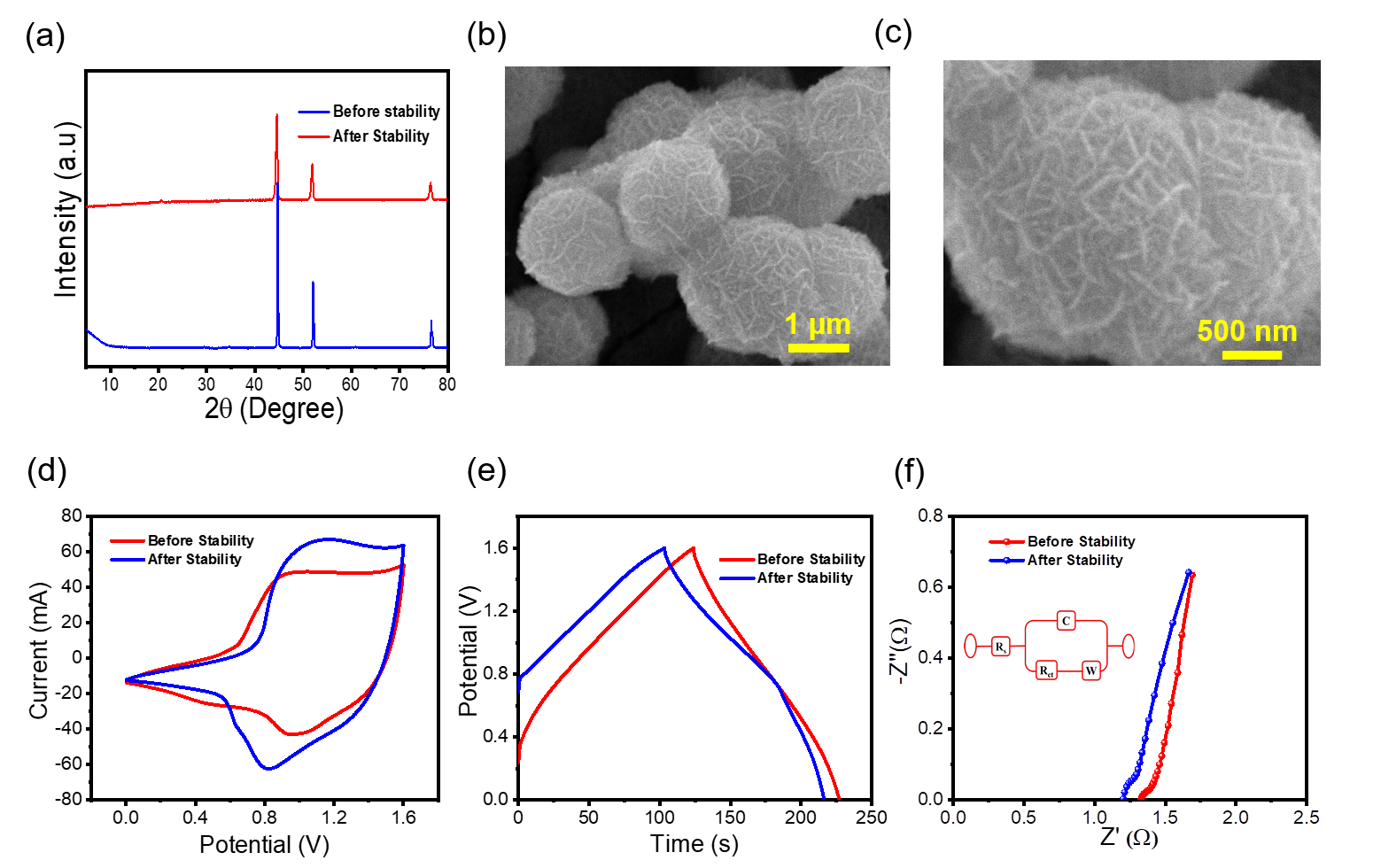
**Fig. S15** (a) Comparative XRD of the before and after stability HSC device samples of NiMoO_4_. (b-c) SEM images of after stability device sample of NiMoO_4_. (d-f) CV, GCD and EIS study of NiMoO_4_//AC HSC device before and after stability over 15000 GCD cycles.

**Symmetric device of NiMoO_4_//NiMoO_4_ testing**

The symmetric NiMoO_4_//NiMoO_4_ device is fabricated for an energy storage device where both the anode and cathode are made of an optimized NiMoO_4_ 6hr sample. We have tested this device in an aqueous 2M KOH solution to evaluate its electrochemical performance in a strongly alkaline environment. The KOH acts as the electrolyte, facilitating the movement of ions between the electrodes during charging and discharging cycles. The use of identical NiMoO_4_ electrodes can simplify the design and improve the overall symmetry and performance of the device. The main objective is to explore the potential of NiMoO_4_ as an electrode material of symmetric device. The CV plot for the NiMoO_4_//NiMoO_4_ symmetric device tested in aqueous 2M KOH solution at different potential windows such as 0.8-1.7 V, highlights its electrochemical performance across varying voltages is shown in **Fig. S16**. As the potential window expands to higher voltages up to 1.7 V, the curves show a more pronounced increase in current, particularly at the positive end of the potential range. This suggests enhanced redox activity of the NiMoO_4_ electrodes, contributing to higher pseudocapacitance. The increase in current with the widening potential window indicates the ability of the device to store more energy. **Fig. S17(a)** shows CV plot for the electrochemical performance of a symmetric NiMoO_4_//NiMoO_4_ device tested at different scan rates ranging from 5-100 mV s^-1^. The CV curves indicating good capacitive behavior with efficient charge storage and release. The increase in current with higher scan rates suggests that the device has a good rate capability and can handle higher power demands ^[7]^. The charge-discharge performance of a symmetric NiMoO_4_//NiMoO_4_ device display in **Fig. S17(b)** evaluated at various constant current densities ranging from 6-10 mA cm^-2^. The charge-discharge profiles across different current densities suggest good capacitive behavior with minimal internal resistance and stable electrochemical reactions. Notably, the device exhibits longer discharge times at lower current densities indicative of higher energy storage capacitance of 49.8 F g^-1^ at current density of 6 mA cm^-2^ is shown in **Fig. S17(c)**, reflects its ability to deliver energy quickly, suitable for high-power applications. The consistent performance across varying currents underscores the robust electrochemical stability and high reversibility of the NiMoO_4_ electrodes in the alkaline electrolyte, making them promising candidates for efficient energy storage devices ^[8]^. The symmetric NiMoO_4_//NiMoO_4_ device observed energy density of 20 Wh kg^-1^ at corresponding power density of 1416.6 W kg^-1^ at current density of 6 mA cm^-2^ is depicted in Ragone plot **Fig. S17(d)**. Noticeably, it still delivered higher power density at 2361.1 W kg^-^1 at energy density of 6.3 Wh kg^-1^ 10 mA cm^-2^. TheNiMoO_4_//NiMoO_4_ device displayed substantial cyclic stability of 122% and columbic efficiency of 97% over 12000 charge discharging cycles in **Fig. S17(e)**. Inset of **Fig. S17(e**) shows the initial and final 5 charging discharging cycles of NiMoO_4_//NiMoO_4_ device at current density of 15 mA cm^-2^. **Fig. S17(f)** shows displays the Nyquist plots of the NiMoO_4_//NiMoO_4_ device both before and after stability testing. These plots indicate low values for both solution resistance and charge transfer resistance, demonstrating that the symmetric device possesses excellent electrical conductivity.

**Fig. S16** The CV of Symmetric device within the potential window of 0–1.8 V.

**Fig. S17. (a)** CV curves of the symmetric device recorded at various scan rates (b) GCD curves of the symmetric device recorded at different current densities (6-10 mA cm^-2^), respectively. (c) Variation of specific capacitance as a function of the current density of symmetric device. (d) Ragone plot of symmetric device at different current densities (6-10 mA cm^-2^). (e) cyclic stability and coulombic efficiency of aqueous symmetric device over 12000 continuous GCD cycles. Inset initial and last 5 cycles. (f) Nyquist plot before and after stability over 12000 GCD cycles.

**Reaction growth mechanism:**

The following chemical reaction shows the formation of NiMoO_4_ on nickel foam by hydrothermal method. The Eq^n^ (S7) shows the precursor Ni(No_3_)_2_.6H_2_O was dissolved in DDW,$\text{Ni}_{(aq)}^{2+}\text{ }$,$\text{2NO}_{3(aq)}^{-}$ ions were separated. The Eq^n^ (S8) shows the precursors of Na_2_MoO_4_.2H_2_O were dissolved in DDW and $\text{2Na}_{^{(aq)}}^{+}\text{ , MoO}_{4(aq)}^{2-}$ions were separated. After the hydrothermal treatment the NiMoO_4_ were formed on nickel foam at 160℃ at varying time 4, 5, 6 and 7hr shown in Eq^n^ (S9) ^[9]^. Directly deposited NiMoO_4_ on nickel foam substrate were dried at 60℃ for overnight.

**References**

[1] V. T. Chebrolu, B. Balakrishnan, S. Aravindha Raja, H. J. Kim, *Dalton Transactions* **2020**, *49*, 9762.

[2] Q. Hu, W. Li, B. Xiang, X. Zou, J. Hao, M. Deng, Q. Wu, Y. Wang, *Electrochim Acta* **2020**, *337*, 135826.

[3] N. R. Chodankar, H. D. Pham, A. K. Nanjundan, J. F. S. Fernando, K. Jayaramulu, D. Golberg, Y. K. Han, D. P. Dubal, *Small* **2020**, *16*, 2002806.

[4] W. Dong, M. Xie, S. Zhao, Q. Qin, F. Huang, *Materials Science and Engineering: R: Reports* **2023**, *152*, 100713.

[5] N. B. Velhal, J. Ahn, T. H. Yun, T. Kim, M. Gu, J. Kim, C. Yim, *ACS Appl Energy Mater* **2023**, *6*, 7405.

[6] F. Ali, N. R. Khalid, *Ceram Int* **2020**, *46*, 24137.

[7] S. K. Shinde, H. M. Yadav, S. Ramesh, C. Bathula, N. Maile, G. S. Ghodake, H. Dhaygude, D. Y. Kim, *J Mol Liq* **2020**, *299*, 112119.

[8] S. Khalid, C. Cao, L. Wang, Y. Zhu, *Scientific Reports 2016 6:1* **2016**, *6*, 1.

[9] X. Xu, J. Shen, N. Li, M. Ye, *J Alloys Compd* **2014**, *616*, 58.
